# Social Support and Psychological Distress among the Bedouin Arab Elderly in Israel: The Moderating Role of Gender

**DOI:** 10.3390/ijerph19074358

**Published:** 2022-04-05

**Authors:** Sarah Abu-Kaf, Ora Nakash, Tsahi Hayat, Michal Cohen

**Affiliations:** 1 Conflict Management & Resolution Program, Department of Interdisciplinary Studies, Ben-Gurion University of the Negev, P.O. Box 653, Beer Sheva 8410501, Israel; 2 Baruch Ivcher School of Psychology, Interdisciplinary Center (IDC) Herzliya, P.O. Box 167, Herzliya 46150, Israel; onakash@smith.edu (O.N.); 85mich@gmail.com (M.C.); 3 School for Social Work, Smith College, Northampton, MA 01063, USA; 4 Sami Ofer School of Communication, Interdisciplinary Center (IDC) Herzliya, P.O. Box 167, Herzliya 46150, Israel; tsahi.hayat@gmail.com

**Keywords:** psychological distress, instrumental support, perceived social support, elderly, Bedouin Arabs

## Abstract

In Israel, as in other developed countries, mental health problems are common among older adults who are members of disadvantaged ethnic minorities that are experiencing cultural and social changes. The main goals of the current study were: (a) to examine gender differences in the levels of psychological distress and social support among Bedouin elders, and (b) to examine the moderating role of gender in the associations between social support indices and psychological distress. We used a cross-sectional design, and independent *t*-tests and hierarchical linear regression analysis were performed. The study was conducted in homes and in social clubs and community centers for elderly people and involved face-to-face interviews and self-administered questionnaires. A convenience sample of 170 Bedouin Arab elderly people living in Israel participated in the study. Participants completed self-report questionnaires that assessed psychological distress, perceived social support, instrumental social support, and socio-demographic characteristics. Male elders reported lower levels of psychological distress and higher levels of instrumental support. Female elders, who reported low levels of both perceived and instrumental support, also reported higher levels of psychological distress. Among the women, there were significant associations between psychological distress and perceived social support, and instrumental support only when the levels of support were low. This study underscores the moderating role of gender in the associations between different types of social support and psychological distress among elderly people belonging to ethnic and cultural underprivileged minority groups.

## 1. Introduction

Mental health issues such as mood disorders and anxiety are prevalent among elderly people. About 20% of adults aged 60 and over suffer from a mental disorder. The most common mental disorder among elderly people is depression, which affects approximately 7% of the world’s older population. Anxiety disorders affect 3.8% of elderly people [1,2] and are particularly common among those from disadvantaged ethnic minority groups [3,4]. Older women have been found to be vulnerable to high levels of common mental health problems, including depressive and anxiety symptoms, as well as somatic complaints [5,6,7,8]. Research on psychological distress among elderly people belonging to ethnic underprivileged minorities, in general, and Bedouin Arabs, in particular, is still very limited.

Bedouin Arabs in Israel belong to the general Arab minority in that country; they comprise about 17% of the Arab population of Israel [9]. The Bedouin Arabs in the south area number 270,000 and represent about 30% of this population [9]. The Bedouin Arab minority faces enormous difficulties and challenges in different domains of daily life (i.e., social-cultural, financial, and political; [10]). The Bedouin Arab minority is characterized by collectivistic, authoritarian, and patriarchal values [11]. Furthermore, this minority receives a hierarchical order in which gender and age-based inequality are prevalent [10].

Fifty percent of the Bedouin population is under the age of 18 and Bedouin Arab seniors make up about 4% of the total Bedouin Arab minority [9]. These elders suffer from very high levels of poverty and low educational levels (the vast majority of them are illiterate; [9]). In addition, during the last two decades, Bedouin individuals have experienced major changes in social structure and norms, including social norms like respect for the elderly people and power relations (i.e., young and female individuals (instead of elders) acting as the heads of their families [10]). As a consequence of these many challenges and difficulties, there has been a higher prevalence of psychological distress among Bedouin Arab individuals, compared to both the general Arab minority population and general Israeli society [10,12,13].

Our recent study of psychological distress, self-esteem, and perceived discrimination amongst Jewish and Bedouin Arab elderly people revealed higher levels of psychological distress among Bedouin Arabs [14]. Still, research on Bedouin Arabs has been scarce and those studies which have been conducted have mostly focused on young and middle-aged individuals. The current study was intended to test the psychological distress and the use of social-support resources among a vulnerable and under-researched group in Bedouin Arab society—the elderly.

### 1.1. Social Support and Psychological Distress among the Elderly

Social support refers to the information that leads individuals to believe that they are loved, respected, valued, and cared for, and to feel that they are a part of a network of communication and mutual obligation [15]. This resource is an outcome of social activities and interactions that promote individuals’ sense of mastery through the sharing of missions and tasks, instrumental and emotional assistance, and emotional comfort [16].

Social support has been found to act as a buffer against the negative effects of stress, decrease psychological distress, and increase well-being [7,17,18,19,20,21,22,23]. In the research literature, the social-support construct has been defined in different ways: as the number of people in the participant’s network, as an index of overall satisfaction with social support, as the availability of several forms or types of support (e.g., informational/emotional, instrumental/ tangible, and affectionate support), and in terms of positive social interaction. There is strong evidence for the benefits of many types of social support, for both mental and physical health [22,24]. In the current study, social support was measured in terms of instrumental support and perceived emotional support.

Elderly individuals are considered to be a vulnerable population. Advanced impairment of individuals’ levels of functioning as they age results in the loss of adaptive responses to stress and a growing risk of age-associated diseases [25,26]. Aging is frequently accompanied by negative changes in social life and may cause as well a diminishment or loss of social relationships [27]. Longitudinal studies have shown that decreased cognitive abilities and perceived control, as well as increased loneliness, may lead to psychological distress among older people [28,29]. Therefore, social support is a particularly important environmental factor for older people’s mental health [21,30].

Previous research has shown that women are more likely to receive social support than men [31,32,33]. Women have also been found to have more female friends than men do, which gives them a greater range of people to call on for emotional support, and they tend to ask for help in times of need [31,32,33]. There is also some evidence that older people rely on different and smaller social groups (mainly immediate family) for social support [34].

There is significant evidence that those with greater social support have fewer mental health problems than those with less social support [17,35,36]. However, there has been limited research on whether the association between social support and psychological distress varies by age or gender [7]. Given the research mentioned above, it is possible that older women may benefit more from social support than older men do.

Despite the large body of research on social support, research is still very limited on this topic in the Bedouin Arab cultural context. As far as we know, there has been no prior research on social support among Bedouin Arab seniors. Therefore, the present study was designed to examine the levels of psychological distress among male and female Bedouin Arab elderly people in Israel. This study also examined the roles of two different types of social support (i.e., instrumental support and emotional perceived support) in the associations between gender and psychological distress.

### 1.2. The Current Study

This study aimed to examine gender differences in the levels of psychological distress and social support among Bedouin elders (55 years old and older). The current study also aimed to examine the associations between psychological distress, perceived social support, and instrumental social support among Bedouin Arab senior individuals in Israel. In the present study, we are interested in the examination of the moderating role of gender in the associations between social support indices and psychological distress.

In light of those objectives, the following hypotheses were proposed:

1. Gender differences in levels of psychological distress, perceived social support, and instrumental social support were expected. Female elderly participants would report higher levels of psychological distress, perceived social support, and instrumental social support compared to male elderly participants [5,6,7,8,31,32,33].
2. We expected to find negative associations between social-support indices and psychological distress [7,17,18,22,23,35,36].
3. We expected that gender would play a moderating role in the associations between perceived social support and instrumental social support and psychological distress [31,32,33].

## 2. Materials and Methods

### 2.1. Participants and Procedure

A convenience sample of 170 elderly individuals from the Bedouin Arab community in southern Israel participated in this study. We conducted a *post hoc* power analysis to examine the actual power of the current results. For a hierarchical linear regression analysis with eight predictors, power analysis indicated that our sample (*n* = 170) was sufficient for the detection of a medium to a large effect (f^2^ = 0.25) with 0.99 power. Participants were recruited using direct person-to-person solicitation of individuals in social clubs and community centers for elderly people in various locations across the south of Israel during the years 2018–2019. We also used snowball-sampling techniques to increase recruitment. After participants signed an informed consent form, they completed self-report questionnaires in Arabic. The research assistants read the scale items for participants with lower educational levels. The Ethics Committee of the Interdisciplinary Center in Herzliya, Israel, approved this research.

### 2.2. Scales

#### 2.2.1. Demographic Questionnaire

A questionnaire was used to get demographic details from the participants. They were asked about their gender, age, education level, and marital status.

#### 2.2.2. General Health Questionnaire (GHQ-12)

The GHQ-12 [37] is commonly used to assess psychological distress during the last month. This scale includes 12 self-report items which are rated on a Likert scale of 4-points (from 0 to 3). The negative items were recoded from 3 (*always*) to 0 (*never*) while positive items were coded from 0 (*always*) to 3 (*never*). The total score is the sum of the scores in all the items; higher scores indicate more severe psychological distress. The GHQ-12 was used and validated in different countries [38,39]. In the current study, we used the Arabic version of this questionnaire [40]. The overall internal consistency reliability for the scale was good (Cronbach’s α = 0.86).

#### 2.2.3. Multidimensional Scale of Perceived Social Support (MSPSS)

The MSPSS [41] is a 12-item self-report measure that inquires about three dimensions of social relationships: family, friends, and significant other. Answers are given on a 7-point Likert-type scale (1 = *very strongly agree* to 7 = *very strongly disagree*). In the current study, higher scores indicate higher levels of perceived support. Although the MSPSS is commonly used to inquire about different dimensions of social relationships, it also produces a global satisfaction with perceived support score that can be obtained by taking the sum of the three scales [41,42]. The internal consistency of the global satisfaction scores in the current study was good (Cronbach’s α = 0.94).

#### 2.2.4. MOS Social Support Survey

The MOS Social Support Survey [43] is a 20-item scale with five response categories: 1 (*none of the time*) to 5 (*all of the time*). The survey consists of four subscales: emotional/informational support, tangible support, affectionate support and positive social interaction. In the current study, we used only the tangible support subscale, which included four items. Internal consistency in the current study was good (Cronbach’s α = 0.90).

### 2.3. Statistical Analysis

The data analysis was performed using SPSS21 with the PROCESS macro developed by Hayes [44] for addressing moderation hypotheses. Independent *t*-tests were also used to examine differences between men and women in terms of the study variables (i.e., psychological distress, perceived social support, and instrumental support). Differences in marital status between the men and women were examined using the χ^2^ test.

Bivariate correlations between independent and dependent variables were calculated using Pearson’s *r* for continuous variables (i.e., psychological distress, perceived social support, and instrumental support).

To examine the moderating role of gender in the association between different types of support and psychological distress while controlling for age, a hierarchical linear regression analysis was performed. Tests of simple effects were conducted for significant three-way interactions.

## 3. Results

### 3.1. Gender Differences in Socio-Demographics and Major Variables of the Study

Table 1 shows the sample’s socio-demographic information. The ages of the participants range from 55 to 90 years (*M* = 65.08, *SD* = 8.05); the majority of the participants were women (60.6%) and married (54.4%). Among the participants, the men were older and more educated than the women. In addition, the majority of the men were married as compared to about half of the Bedouin Arab women. Whereas, about one-third of women were divorced, separated or widowed compared to 6.5% of men.

Significant gender differences were found in psychological distress, with women reporting greater psychological distress compared to men. In addition, marginally significant gender differences were found in instrumental support, with men reporting greater instrumental support than women. There were no significant gender differences in perceived social support.

### 3.2. The Moderating Role of Gender in the Associations between Types of Support and Psychological Distress

Instrumental support was positively correlated with perceived social support (r = 0.42, *p* < 0.01). There were significant negative correlations between both types of social support and psychological distress (r = −0.38, *p* < 0.01 for instrumental support and r = −0.23, *p* < 0.05 for perceived social support).

To examine the moderating role of gender in the correlations between different types of support and psychological distress while controlling for age, a four-step, hierarchical linear regression analysis was conducted. Psychological distress was entered as the dependent variable. In the first block, age was the only independent variable. Gender, perceived social support, and instrumental support were entered in the second block. The three two-way interactions between gender and each of the two variables of social support were entered in the third block. Finally, in the fourth block, a three-way interaction of gender × perceived social support × instrumental support was entered.

The model of the moderating role of gender in the association between types of support and psychological distress was significant, predicting 25.1% of the variance in psychological distress. As shown in Table 2, only gender and instrumental support were associated with psychological distress, suggesting that being a man (partial r = 0.23, *p* < 0.05) and greater instrumental support (partial r = −0.29, *p* < 0.01) are associated with lower levels of psychological distress.

Most importantly, the analysis revealed a significant three-way interaction effect between gender, perceived social support, and instrumental support (*partial* r = 0.21, *p* < 0.05). Simple slopes for the associations between the interactions of different levels of perceived social support and instrumental support were tested for each gender. Among the women, there were significant associations between the variables only at low levels of perceived social support and instrumental support (*b* = 4.97, *SEb* = 2.18, *β* = 0.32, *p* < 0.05), meaning that Bedouin women who reported low levels of both perceived and instrumental support also reported higher levels of psychological distress. There were no significant correlations between the variables at high levels of support among the women and no correlations at all among the men (all *t* < 1.9, *n.s.*).

## 4. Discussion

The current study was intended to assess gender differences in psychological distress, perceived social support, and instrumental social support among Bedouin Arab elders. In addition, we were interested in evaluating the role played by gender in the associations between social-support indices and psychological distress. The current study yielded several significant findings that are worthy of discussion.

Our results revealed gender differences in the reported levels of psychological distress. Female Bedouin elders reported higher levels of psychological distress than male Bedouin elders. This finding supports previous research that found that women aged 60 or older are more vulnerable to psychological distress than men [5,6,7,8]. The greater psychological distress experienced by the women in our study may have been related to the fact that they had more household responsibilities than the men in our study, in line with the division of household labor that is characteristic of traditional Arab society. In such a cultural and social context, the family structure may induce more stress, which can lead to more psychological distress among these women [45]. For instance, Bedouin Arab senior women have significantly greater caregiving responsibilities for adult children and grandchildren. Moreover, in our female Bedouin sample, about 40% of the participants were single (i.e., divorced, separated, or widowed) and 13.5% were married to a spouse who was married to more than one wife. Elders who are married or in a relationship have been found to report fewer depressive symptoms than those who are divorced or widowed [46]. Polygamous marriage has been associated with greater stress in women’s lives; stress that may be relational (e.g., jealousy and acrimony between the families; [47]) or economic, as polygamous marriages contain greater numbers of children, but do not necessarily have correspondingly greater incomes than monogamous family units [48,49]. Polygamous women reported greater psychological distress, more experiences of loneliness, lower self-esteem, and more hostility, paranoid ideations, anxiety, depression and somatic complaints [49,50,51]. In addition, the vast majority of Bedouin elder women have very low levels of education and employment [13,52], which may put additional pressure and increase their risk for the development of psychological distress. In addition, a recent study conducted by Edelstein et al. [53] found the lowest engagement in leisure/recreation-related physical activity was accompanied by less supportive norms’ attitudes toward those activities among Bedouin Arab elder women. Leisure/recreation-related physical activity, but not work-related physical activity, has been found to decrease psychological distress [54,55]. Furthermore, previous studies have suggested that work-related physical activity may be associated with higher levels of psychological distress [54].

Interestingly, we found gender differences in the levels of instrumental support. The older men reported higher levels of instrumental support than the older women. This finding does not support previous research, which found that women are more likely than men to receive social support [31,32,33]. This finding may be explained by the sex roles and the division of labor in Arab society, in general, and Bedouin Arab society, in particular, in which it is commonly accepted that women are the main caregivers who should look after family members, particularly those who suffer from physical or mental illnesses, and women’s health is a lower priority than that of other family members [56,57]. Bedouin Arab elder women receive more attention and support from their families and have fewer caregiver obligations than younger Bedouin women. However, they are still expected to take care of their aging husbands. In recent decades, many young Bedouin Arabs have become involved in higher-education studies and have integrated into the job market, but there is still a lack of pre-schools in Bedouin Arab society [58]. In this context, many elder Bedouin women have become the main caregivers for their grandchildren. This position puts more stress and greater responsibilities on the shoulders of these women. In this context, older Bedouin women give more and receive less instrumental support from other family members.

Importantly, among Bedouin female elders, lower levels of perceived social and instrumental support were related to higher levels of psychological distress, but higher levels of that support were not. In contrast, none of these variables were found to be related to psychological distress among Bedouin male elders. This finding is consistent with previous studies that have found that women, in general, and female elders, in particular, rely more on social support than males do [31,32,33], as well as research that found that women benefit more from social support than men do [7]. The importance of social networks in the context of health issues among older Bedouin women was addressed by Edelstein et al. [59]. Those researchers claimed that limited and weaker social networks may limit these women’s exposure to health-related knowledge, which may lead to poorer health [60]. The current study has added to this body of research, in that it revealed that women who experience lower levels (or no) social support (both instrumental and perceived) may be more likely to experience greater psychological distress.

The present study has some limitations and there are areas that require further examination in future studies. At first, our data was collected from a female-dominated convenience sample of 97 females and 73 males. Future studies should include larger samples with more male participants. Second, the current study included more single female elders (i.e., divorced, separated, or widowed) than single male elders. Marital status may have implications on our study variables (i.e., social support and psychological distress). Moreover, education is an important confounding variable in studies examining mental health distress. However, the distribution of the formal education of the participants in the current study was skewed, such that over 50% had 0 years of formal education. Therefore, we were not able to use years of formal education as a control variable in the regression model. Future research should control for those demographic variables by recruiting similar samples from different marital statuses and education-level categories. Future studies should also include variables related to different types of violence, which may induce significant aggravation of psychological stress among elderly people, particularly elderly women [61,62].

Finally, future studies should include measures for different sources of stress among elders who are living in multi-generational households [45].

## 5. Conclusions

The importance of this study lies in its examination of the levels of psychological distress and two types of social support among male and female Bedouin Arab elders, who are considered to be part of a disadvantaged ethnic minority and an under-researched population. The present study highlights the vulnerability of female Bedouin elders to psychological distress, particularly in the context of the lower levels of social support that they receive.

The results of this study suggest that mental health professionals who work with the elderly in Bedouin Arab society should design and implement interventions that focus on the Bedouin Arab elderly as a vulnerable group for the development of psychological distress. Professionals working with ethnic-minority elders should pay more attention to elder women and be aware of the importance and the benefits of social support for those women. Intervention programs should assist elder women by providing them with group support, to increase the chances of those women feeling more strongly affiliated with and belonging to a social network of other women (through the experience of being involved in relationships with significant others, particularly women from the same cultural background). Moreover, those elder women should be integrated into meaningful and enjoyable social activities, which should help to relieve the stress that they experience in association with their responsibilities and improve their levels of functioning, all of which may have significant value for preventing future psychological distress.

## Figures and Tables

**Table 1:** Socio-demographics and major variables of the study by gender.

	Women (*N* = 97)	Men (*N* = 73)	Statistic
Age	63.56 (6.92)	67.42 (9.10)	*t* (107.45) = 2.87, *p* < 0.01
Education ^a^	2.21 (3.38)	5.47 (5.46)	*t* (96.36) = 3.89, *p* < 0.001
Marital status ^a^			*χ^2^*(4) *=* 8.99, *p* < 0.01
Single	2 (2.1%)	1 (1.6%)	
Married	44 (45.8%)	43 (69.4%)	
Divorced/separated	10 (10.5%)	0 (0%)	
Widowed	27 (28.1%)	4 (6.5%)	
Married with more than one wife (for men)	-	14 (22.6%)	
Married to a man who is married with more than one wife (for women)	13 (13.5%)	-	
Perceived social support ^a^	63.48 (18.02)	62.06 (18.93)	*t* (115) = −0.41, *n.s.*
Instrumental support ^a^	72.41 (28.73)	81.91 (25.41)	*t* (115) = 1.83, *p* = 0.07
Psychological distress ^a^	26.74 (7.70)	23.82 (7.23)	*t* (156) = −2.38, *p* < 0.05

^a^ The total number of responses is smaller than the sample size (*N* = 170) due to missing responses to these questions. n.s.= non-significant.

**Table 2:** Hierarchical linear model of the moderating role of gender in the associations between both types of support and psychological distress.

	B	*SE*	*β*
Step 1			
Age	0.08	0.08	0.09
Step 2			
Gender	2.94	1.34	0.19 *
Perceived social support	−0.03	0.04	−0.07
Instrumental support	−0.08	0.03	−0.30 **
Step 3			
Gender × Perceived social support	−0.03	0.08	−0.18
Gender × Instrumental support	−0.05	0.06	−0.40
Perceived social support × Instrumental support	0.00	0.00	0.18
Step 4			
Gender × Perceived social support × Instrumental support	0.01	0.00	0.21 *

* *p* < 0.05; ** *p* < 0.01.

## Data Availability

The datasets used and/or analyzed during the current study are available from the corresponding author on reasonable request.

## References

[1] Parkar S.R. (2015). Elderly Mental Health: Needs. Mens Sana Monogr..

[2] World Health Organization (WHO) (2017). Depression and Other Common Mental Disorders: Global Health Estimates.

[3] Garrett M.D., Baldridge D., Benson W., Crowder J., Aldrich N. (2015). Mental Health Disorders among an Invisible Minority: Depression and Dementia among American Indian and Alaska Native Elders. Gerontology.

[4] Willging C.E., Sommerfeld D.H., Jaramillo E.T., Lujan E., Bly R.S., Debenport E.K., Verney S.P., Lujan R. (2018). Improving Native American Elder Access to and Use of Health Care through Effective Health-System Navigation. BMC Health Serv. Res..

[5] Girgus J.S., Yang K., Ferri C.V. (2017). The Gender Difference in Depression: Are Elderly Women at Greater Risk for Depression than Elderly Men?. Geriatrics.

[6] Gronning K., Espnes G.A., Nguyen C., Rodrigues A.M.F., Gregorio M.J., Sousa R., Canhão H., André B. (2018). Psychological Distress in Elderly People Is Associated with Diet, Wellbeing, Health Status, Social Support and Physical Functioning—A HUNT3 Study. BMC Geriatr..

[7] Milner A., Krnjacki L., LaMontagne A.D. (2016). Age and Gender Differences in the Influence of Social Support on Mental Health: A Longitudinal Fixed-Effects Analysis Using 13 Annual Waves of the HILDA Cohort. Public Health.

[8] Perkins J.M., Lee H.-Y., James K.S., Oh J., Krishna A., Heo J., Lee J.-K., Subramanian S.V. (2016). Marital Status, Widowhood Duration, Gender and Health Outcomes: A Cross-Sectional Study among Older Adults in India. BMC Public Health.

[9] Central Bureau of Statistics, State of Israel (2020). Statistical Abstract of Israel. http://www.cbs.gov.il.

[10] Abu-Kaf S., Haj-Yahia M., Nakash O., Levav I. (2019). Mental Health Issues among Arab Palestinian Women in Israel. Mental Health and Palestinian Citizens in Israel.

[11] Peleg-Popko O., Klingman A., Abu-Hanna Nahhas I. (2003). Cross-Cultural and Familial Differences between Arab and Jewish Adolescents in Test Anxiety. Int. J. Intercult. Relat..

[12] Abu-Kaf S., Shahar G. (2017). Depression and Somatic Symptoms among Two Ethnic Groups in Israel: Testing Three Theoretical Models. Isr. J. Psychiatr. Relat. Sci..

[13] Braun-Lewensohn O., Abu-Kaf S., Al-Said K., Huss E. (2019). Analysis of the Differential Relationship between the Perception of One’s Life and Coping Resources among Three Generations of Bedouin Women. Int. J. Environ. Res. Public Health.

[14] Abu-Kaf S., Nakash O., Hayat T., Cohen M. (2020). Psychological distress among the Bedouin Arab and Jewish Elderly in Israel: The Roles of Gender, Discrimination, and Self-Esteem. Psychiatr. Res..

[15] Cobb S. (1976). Social Support as a Moderator of Life stress. Psychosom. Med..

[16] Caplan G. (1974). Support Systems and Community Mental Health: Lectures on Concept Development.

[17] Cohen S., Wills T.A. (1985). Stress, Social Support, and the Buffering Hypothesis. Psychol. Bull..

[18] Dalgard O.S., Thapa S.B., Hauff E., McCubbin M., Syed H.R. (2006). Immigration, Lack of Control and Psychological Distress: Findings from the Oslo Health Study. Scand. J. Psychol..

[19] Gleason M., Iida M., Mikulincer M., Shaver P.R. (2015). Social Support. APA Handbook of Personality and Social Psychology.

[20] Harandi T.F., Taghinasab M.M., Nayeri T.D. (2017). The Correlation of Social Support with Mental Health: A Meta-Analysis. Electron. Physician.

[21] Smyth N., Siriwardhana C., Hotopf M., Hatch S.L. (2015). Social Networks, Social Support and Psychiatric Symptoms: Social Determinants and Associations within a Multicultural Community Population. Soc. Psychiatr. Psychiatr. Epidemiol..

[22] Thoits P.A. (1995). Stress, Coping, and Social Support Processes: Where Are We? What’s Next?. J. Health Soc. Behav..

[23] Uchino B.N. (2004). Social Support and Physical Health: Understanding the Health Consequences of Relationships.

[24] Rodriguez N., Mira C.B., Myers H.E., Monis J.K., Cardoza D. (2003). Family or Friends: Who Plays a Greater Supportive Role for Latino College Students?. Cult. Divers. Ethn. Minority Psychol..

[25] Chen Y., Hicks A., While A.E. (2014). Loneliness and Social Support of Older People in China: A Systematic Literature Review. Health Soc. Care Community.

[26] Ren Z. (2002). The Life and Health Status of Urban Elderly People in Bejing, China. Master’s Thesis.

[27] Chalise H.N., Saito T., Takahashi M., Kai I. (2007). Relationship Specialization amongst Sources and Receivers of Social Support and Its Correlations with Loneliness and Subjective Well-Being: A Cross-Sectional Study of Nepalese Older Adults. Archiv. Gerontol. Geriatr..

[28] Wagner J., Gerstorf D., Hoppmann C., Luszcz M.A. (2013). The Nature and Correlates of Self-Esteem Trajectories in Late Life. J. Pers. Soc. Psychol..

[29] Wagner J., Hoppmann C., Ram N., Gerstorf D. (2015). Self-Esteem is Relatively Stable Late in Life: The Role of Resources in the Health, Self-Regulation, and Social Domains. Dev. Psychol..

[30] Yeh S.C.J., Lo S.K. (2004). Living Alone, Social Support, and Feeling Lonely among the Elderly. Soc. Behav. Pers. Int. J..

[31] Liebler C.A., Sandefur G.D. (2002). Gender Differences in the Exchange of Social Support with Friends, Neighbors, and Co-Workers at Midlife. Soc. Sci. Res..

[32] Nolen-Hoeksema S., Hilt L.M., Nolen-Hoeksema S., Hilt L.M. (2009). Gender Differences in Depression. Handbook of Depression in Adolescents.

[33] Taylor S.E., Klein L.C., Lewis B.P., Gruenewald T.L., Gurung R.A.R., Updegraff J.A. (2000). Biobehavioral Responses to Stress in Females: Tend-and-Befriend, Not Fight-or-Flight. Psychol. Rev..

[34] Cheng S.T., Chan A.C.M. (2004). The Multidimensional Scale of Perceived Social Support: Dimensionality and Age and Gender Differences in Adolescents. Pers. Individ. Diff..

[35] Kawachi I., Berkman L.F. (2001). Social Ties and Mental Health. J. Urb. Health.

[36] Lakey B., Orehek E. (2011). Relational Regulation Theory: A New Approach to Explain the Link between Perceived Social Support and Mental Health. Psychol. Rev..

[37] Goldberg D. (1978). Manual of the General Health Questionnaire.

[38] Kessler R.C., Ustun T.B. (2008). Global Perspectives on the Epidemiology of Mental Disorders. The WHO Mental Health Surveys.

[39] Nakash O., Nagar M., Danilovich E., Bentov-Gofrit D., Lurie I., Steiner E., Sadeh-Sharvit S., Szor H., Levav I. (2014). Ethnic Disparities in Mental Health Treatment Gap in a Community-Based Survey and in Access to Care in Psychiatric Clinics. Int. J. Soc. Psychiatr..

[40] Nakash N., Hayat T., Abu-Kaf S., Cohen M. (2019). Association between Knowledge about How to Search for Mental Health Information and Psychological distress among Older Adults: The Moderating Role of Immigration Status. J. Gerontol. Soc. Work.

[41] Zimet G.D., Dahlem N.W., Zimet S.G., Farley G.K. (1988). The Multidimensional Scale of Perceived Social Support. J. Pers. Assess..

[42] Clara I., Cox B., Enns M., Murray L., Torgrudc L. (2003). Confirmatory Factor Analysis of the Multidimensional Scale of Perceived Social Support in Clinically Distressed and Student Samples. J. Pers. Assess..

[43] Sherbourne C.D., Stewart A.L. (1991). The MOS Social Support Survey. Soc. Sci. Med..

[44] Hayes A.F. (2012). PROCESS: A Versatile Computational Tool for Observed Variable Mediation, Moderation, and Conditional Process Modeling. http://www.afhayes.com/public/process2012.pdf.

[45] Takeda Y., Kawachi I., Yamagata Z., Hashimoto S., Matsumura Y., Oguri S., Okayama A. (2004). Multigenerational Family Structure in Japanese Society: Impacts on Stress and Health Behaviors among Women and Men. Soc. Sci. Med..

[46] Barger S.D., Messerli-Burgy N., Barth J. (2014). Social Relationship Correlates of Major Depressive Disorder and Depressive Symptoms in Switzerland: Nationally Representative Cross-Sectional Study. BMC Public Health.

[47] Al-Krenawi A. (1999). Explanations of Mental Health Symptoms by the Bedouin-Arabs of the Negev. Int. J. Soc. Psychiatr..

[48] Al-Krenawi A. (1998). Family Therapy with a Multiparental/Multispousal Family. Fam. Process.

[49] Al-Krenawi A., Graham J.R. (2006). A Comparison of Family Functioning, Life and Marital Satisfaction, and Mental Health of Women in Polygamous and Monogamous Marriages. Int. J. Soc. Psychiatr..

[50] Al-Krenawi A. (2001). Women from Polygamous and Monogamous Marriages in an Out-Patient Psychiatric Clinic. Transcult. Psychiatr..

[51] Daoud N., Braun-Lewensohn O., Eriksson M., Sagy S. (2014). Sense of Coherence and Depressive Symptoms among Low-Income Bedouin Women in the Negev Israel. J. Ment. Health.

[52] Farjoon B. (2018). A Look at the Employment of Bedouin Arab Women in the Negev.

[53] Edelstein O.E., Vered I., Sarid O. (2021). Correlates of Participation in Physical Activity among Older Women in Israel: Does Ethno-Cultural Background Matter?. Health Promot. Int..

[54] Mumba M.N., Nacarrow A.F., Cody S., Key B.A., Wang H., Robb M., Jurczyk A., Ford C., Kelley M.A., Allen R.S. (2020). Intensity and Type of Physical Activity Predicts Depression in Older Adults. Aging Ment. Health.

[55] Palmlof L., Holm L.W., Alfredsson L., Magnusson C., Vingard E., Skillgate E. (2016). The Impact of Work-Related Physical Activity and Leisure Physical Activity on the Risk and Prognosis of Neck Pain—A Population-Based Cohort Study on Workers. BMC Musculoskel. Disord..

[56] Douki S., Zineb S.B., Nacef F., Halbreich U. (2007). Women’s Mental Health in the Muslim World: Cultural, Religious, and Social Issues. J. Affect. Disord..

[57] Reinhardt U., Cheng T. (2000). The world health report 2000—Health systems: Improving performance. Bull. World Health Organ..

[58] State Comptroller (Israel) (2015). Report on the Implementation of the Education Reform and Reducing the Gaps in Early Childhood Education. http://www.mevaker.gov.il/he/Reports/Report_290/ReportFiles/fullreport_2.pdf.

[59] Edelstein O.E., Achdut N., Vered I., Sarid O. (2020). Determinants of Bone Mineral Screening Behavior among Three Ethno-Cultural Groups of Women in Israel. Int. J. Environ. Res. Public Health.

[60] Masic I., Sivic S., Toromanovic S., Borojevic T., Pandza H. (2012). Social Networks in Improvement of Health Care. Mat. Soc. Med..

[61] World Health Organization (WHO) (2021). Elder Abuse. Fact Sheet. https://www.who.int/news-room/factsheets/detail/elder-abuse.

[62] Yon Y., Mikton C.R., Gassoumis Z.D., Wilber K.H. (2017). Elder Abuse Prevalence in Community Settings: A Systematic Review and Meta-Analysis. Lancet Glob. Health.

